# Everyday Functioning Benefits from an Assisted Living Platform amongst Frail Older Adults and Their Caregivers

**DOI:** 10.3389/fnagi.2017.00302

**Published:** 2017-09-28

**Authors:** Lucile Dupuy, Charlotte Froger, Charles Consel, Hélène Sauzéon

**Affiliations:** ^1^Phoenix Team Project, Inria, Talence, France; ^2^Laboratoire Handicap, Activité, Cognition et Santé (EA 4136), University of Bordeaux, Bordeaux, France; ^3^Bordeaux-National Institute of Technology, Talence, France

**Keywords:** aging, frailty, everyday functioning, caregiver burden, ambient assisted living technologies

## Abstract

Ambient assisted living technologies (AAL) are regarded as a promising solution to support aging in place. Yet, their efficacy has to be demonstrated in terms of benefits for independent living and for work conditions of caregivers. Hence, the purpose of this study was to assess the benefits of a multi-task AAL platform for both Frail older Individuals (FIs) and professional caregivers with respect to everyday functioning and caregiver burden. In this context, a 6-month field study involved 32 FIs living at home (half of them were equipped by the platform and the remaining half were not, as a control condition) and their caregivers. Everyday functioning measures were reported by frail participants and caregivers. Self-reported burden measures of caregiver were also collected. The main results showed that the caregiver's estimates of everyday functioning of equipped participants were unchanged across time, while they decreased for the control participants. Also, a reduction of self-reported objective burden was obtained after 6 months of AAL intervention for the equipped group, compared to the control group. Overall, these results highlighted the potential of AAL as a relevant environmental support for preventing both functional losses in FIs and objective burden professional caregiver.

## Introduction

Over the last decade, extensive research efforts have been provided to develop technologies that support aging in place, and that reduce caregiver burden. Current solutions include a variety of assistive technologies that were developed according to the “ambient intelligence” paradigm. This paradigm aims to empower people's capabilities by means of digital environments that are sensitive, adaptive, and responsive to human needs (Rashidi and Mihailidis, 2013 for review). These assisted living technologies are thus called ambient-assisted living (AAL) devices. AAL devices are thought to provide home safety for the elderly, help with daily activities, and promote older adults' social participation by increasing connection and communication with their social network (Rashidi and Mihailidis, 2013). However, a common drawback in existing AAL technologies is the lack of experimental validation (Reeder et al., 2013). In this vein, the benefits of AAL should be demonstrated with respect to both the autonomy of older adults, and the caregiver's self-perceived burden. Consequently, the purpose of the present study was to evaluate the long-term use of an AAL platform named HomeAssist (Dupuy et al., 2016). The expected outcomes were the promotion of independent living and the reduction of the caregiver burden. More particularly, an experimental field study was conducted in which HomeAssist was deployed in a real setting: the home of frail, community-dwelling older adults. The pilot field study included 32 older adults (half of them were equipped with HomeAssist) and their professional caregivers, and a 6-month follow-up.

Frailty is a common and important geriatric syndrome characterized by an age-related decline in physical, cognitive and physiological function, leading to increased vulnerability to adverse health outcomes, including death, hospitalization, disability and age-related dementias (Clegg et al., 2013; for review)^1^. According to the phenotypic definition of frailty proposed by Fried et al. (2001), frailty refers to individuals who meet three or more of the five following criteria: weakness, slowness, low level of physical activity, self-reported exhaustion, and unintentional weight loss.

As a result, Frail Individuals (FIs) are at high risk of losing their autonomy for everyday functioning (Ávila-Funes et al., 2008). Also, FIs are acknowledged to be an optimal target population for the implementation of dependency prevention programs (Cesari et al., 2014). Moreover, there is increasing evidence to suggest that environmental support can be effective for helping FIs to perform everyday activities, or even to reduce their functional degradation (e.g., first controlled trial: Mann et al., 1999). According to the Environmental Support framework for aging (Craik, 1986), assistive devices for ADLs refer to all instruments that either provide an adaptation of the environment to make it more accommodating (i.e., to reduce the demands of a given task), or that equip people with the means to compensate for their impairments (i.e., to support the use of a person's resources) (Morrow and Rogers, 2008).

Caregivers are important resources for FIs, acting as “human environmental support for ADL” (e.g., Gillespie et al., 2012). These caregivers prompt, remind and provide support for the performance of everyday activities. However, this form of assistance also creates problems for both caregivers (i.e., burden) and older adults (Lopez-Hartmann et al., 2012 for review).

### Assessment of older adults' functional status and caregiver burden

Independent everyday functioning, also called functional status, refers to an individual's abilities to autonomously perform basic (BADL) and instrumental (IADL) activities of daily living. BADLs correspond to physical self-care tasks, such as dressing and toileting (Katz, 1983). IADLs entail more cognitively complex tasks, including meal preparation or medication management (Lawton and Brody, 1969). In general, functional status is assessed through self-report questionnaires. However, it has been shown that older adults tend to underestimate their everyday difficulties, whereas caregivers are more accurate in assessing older adults' functional status (e.g., Gold, 2012). Thus, self-report questionnaires are increasingly been complemented with caregiver-reported questionnaires. These questionnaires include for instance the Caregiver's Perceptions of Functional Status Scale (Loewenstein et al., 1989) and the validated French questionnaire *Inventaire des Habiletés pour la Vie en Appartement* (IHVA, Corbeil et al., 2009, for the original English version, see the Scale of independent behavior revised, Bruininks et al., 1996). The IHVA has the advantage of assessing everyday abilities within 12 dimensions, including health management, meal preparation, and community capabilities.

Caregiver burden can be defined as the experience of “enduring stress and frustration” by those who care for individuals with reduced autonomy (e.g., Zarit et al., 1980). Caregivers can be informal (e.g., family or close friends) or professional (e.g., nurses or home care professionals). Therefore, questionnaires that assess caregiver burden are designed to suit either professional or informal caregivers. The most widely used questionnaires are the Zarit Burden Inventory (Zarit et al., 1980) for informal caregiver assessments; and the Maslach Burnout Inventory (MBI - Maslach et al., 1997) for professional caregivers.

### Ambient-assisted living tools for older adults and their caregivers

AAL tools for older adults can be divided into three categories, according to the person's needs growing up with senescence: everyday activities, home safety, and social participation (Baecker et al., 2012; Huber et al., 2013). As reported in Dupuy et al. (2015), AAL devices for everyday activities include digital pillboxes, electronic organizers for managing appointments, and tools for monitoring daily activities, which can supply users with notifications should they forget something. Home safety AAL devices mainly focus on the prevention of falls and common domestic accidents, by means of fall detectors, lighting path and alarms for caregivers. Finally, AAL systems can deliver specific social functionalities, which consist of social gaming technologies, simplified electronic mailing, video telephoning, and digital picture frames.

Ambient assisted living technologies devices can also be an efficient means for reducing interpersonal tension between caregivers and care-receivers, in addition to increasing the quality of care (for a review, see Chi and Demiris, 2015). Such services include for instance videophones, phone-based systems, and web-based information.

Unfortunately, the growing supply of AAL for aging in place does not translate into technology adoption by older adults (Peek et al., 2014). As a result, researchers in the field of Aging and Human Factors have investigated the factors affecting technology acceptance amongst the older adults. According to the Senior Technology Acceptance Model (Chen and Chan, 2014], and previous related studies, three main families of factors are identified as barriers or assets of technology acceptance: (1) the characteristics of older persons (e.g., perceived needs, technological skills, medical conditions), (2) their environment (e.g., social support for using technologies, living place), (3) the features of technology (e.g., hardware, interface accessibility, usability) (e.g., for reviews, Peek et al., 2014; Queirós et al., 2015).

Despite considerable efforts for leveraging the knowledge on aging and human factors, several AAL-related issues remain to be resolved. First, their silo-based nature makes it a challenge to aggregate them. Indeed, older adults require more and more services to assist an increasing number of ADLs due to multiple, various and evolving needs, particularly in the context of frailty. As a result, personalized multiple intervention programs are more efficient (and sometimes less costly) to slow the impact of frailty (on cognition, autonomy, quality of life) than a usual intervention program (Fairhall et al., 2015). Second, the silo-based nature of AAL devices generates an overwhelming cognitive cost for older users, as documented in the literature on aging (Fisk et al., 2009). A third limitation is related to the contextual relevance of assistive services (i.e., situation/context awareness). Indeed, most AAL devices rely on an isolated telecommunication system (Chi and Demiris, 2015). Thus, such services are not flexible and are supplied irrespective of a person's actual needs for a given situation, rendering them unsuitable, or indeed even obstructive for performing ADLs.

To overcome these limitations, AAL is increasingly based on smart homes (SH) (for reviews, Tomita et al., 2010; Morris et al., 2013). A SH can be defined as a regular home, augmented with various types of sensors that can be used to supply multiple assistive services (Rashidi and Mihailidis, 2013). Today, most of these SH are implemented in laboratory settings or in dedicated communities (Tomita et al., 2010). Hence, the greatest limitation of such solutions is that older adults must be moved out of their familiar environment, impeding their regular everyday functioning. Very few projects have addressed the issue of retrofitting existing homes to turn them into SH (i.e., adding sensors and assistive technologies in older adults' own house) (Tomita et al., 2007; Cook et al., 2013; Rafferty et al., 2017). This approach has the advantage of being less disruptive for older adults to perform their daily routines. Surprisingly, there is little evidence of the efficacy of such retrofitting-based SH in terms of benefits for the older adult's autonomy and caregiver burden.

### Existing experimental study on health benefits from AAL

Among the increasing amount of AAL tools for aging in place, only few have been validated in an experimental study. A recent systematic review (Liu et al., 2016) highlighted that only 33.33% of the reviewed studies investigated the users' benefits from AAL; and only 18.75% included a control group for assessing AAL efficacy.

Among the available studies, the work of Tomita et al. (2007) is of particular interest. In this study, the homes of 46 older adults who lived alone were retrofitted using X10 products (a technology based on wired sensors) and a computer. The 46 users were compared over a 2-year period with 67 control older adults. The measurements taken in this study were diverse and included standardized clinical assessments of functional status, cognitive status, health conditions, and physical abilities. Results indicated no change in functional, physical and cognitive measures in the intervention group, whereas the control group declined significantly in each collected measure. Another interesting study is that conducted by Vincent and his collaborators (Vincent et al., 2006), which evaluated the impact of a tele-surveillance system on the wellbeing of older adults. More specifically, they gathered measures of cognitive and functional status, and perceived quality of life (using standardized clinical assessments), and the number of days spent in hospital over a 6-month period for 38 older adults using the system. Results showed no effect on the quality of life and functional status of the older adults, but they did reveal a great reduction in the length of hospital stays. However, this study did not include a control group.

Regarding the caregivers, studies have very rarely evaluated the benefits of AAL for reducing their burden. Nevertheless, the above-mentioned study by Vincent et al. (2006) also included 38 informal caregivers, and measured the impact of the tele-surveillance system on their burden (assessed with a “hand-made” scale). Results showed a decrease of burden after 6 months using the system. In this vein, Magnusson and Hanson (2005) conducted a qualitative study with 34 families, in which professional practitioners and families were interviewed regarding a communication platform for older adults. The platform consisted in a television-based technology providing multi-media programs and videophone facility for caregivers, and was deployed in older adults' home for approximately 3 months. Feedback from interviews with caregivers highlighted the positive feeling of such a technology in reducing caregiver burden and improving caregivers' satisfaction.

Overall, AAL research appears as suffering from several drawbacks: first, AAL devices used were relatively simple (remotely controlled sensors, call pendants, phones) and mostly single-task regarding the three domains of assistance (i.e., everyday activities, safety or social participation); second, studies often fail to use methodological standards (no control conditions, or “hand-made” scales); and finally, some studies failed to consider the caregivers.

Consequently, we developed an AAL platform, namely HomeAssist that consisted of assistive applications belonging to the three domains of assistance: everyday activities, safety, and social participation. HomeAssist benefits were assessed in a 6-month experimental study. Participants consisted of two matched groups (equipped vs. control groups). Expected results were positive, duration-dependent effect of the platform on FIs' functional status (whether by improving or maintaining everyday functioning, or at least slowing a potential decline), compared to the control group. As well, we expected a positive impact on caregiver burden for the equipped group.

## Methods

This section presents the experimental validation of our platform, by assessing the benefits for older adults and their caregivers. HomeAssist was deployed in the house of 16 community-dwelling older adults for a 6-month period. We also recruited 16 matched older adults to form a control group. Furthermore, a professional caregiver for each of our 32 participants was also included in the experimental study. First, the participants and the HomeAssist platform are described. The assessments used throughout the intervention are detailed afterwards.

### Participants

We recruited 32 dyads, each comprising one older adult and his/her professional caregiver. Participants were recruited thanks to collaborations with public home care services for community-dwelling older adults. We selected cognitively healthy oldest old participants (MMSE, Folstein et al., 1975, with a score greater than 25; Lechevallier-Michel et al., 2004), living alone and older than 70 years of age (from 70 to 90).

All the participants were native French speakers. They underwent a geriatric assessment to evaluate frailty dimensions, according to Fried et al. (2001). First, physical reserve was assessed using several tasks selected from widely used clinical and research scales (assessing weight, weakness, slowness, and low level of physical activity) as follows:

-The Mini Nutritional Assessment (Guigoz and Vellas, 1999) and the extraction of *Body mass* and *lean mass values*^2^ as important components of frailty (Campbell and Buchner, 1997). This enabled a lean body mass score, ranging from 0 to 5, to be calculated.
-*Static Balance Testing* (from the SPPB - Short Physical Performance Battery, Guralnik et al., 2000) consists of three sorts of standing: side-by-side, semi-tandem and tandem stand; scored from 4–the participant holds the tandem position for more than 10 s; to 0–the participant did not attempted any standing position.
-*Timed Get Up and Go Test* (Podsiadlo and Richardson, 1991) consists in rising from a chair, walking three meters, turning around, walking back to the chair, and sitting down. Time in seconds to complete the task is recorded and scored as followed: 1–the task is completed in more than 30 s, 2–the task is completed from 20 to 30 s, and 3–the task is completed in less than 10 s (in this case, mobility is considered normal).
-*Gait Speed Test* (from the SPPB) corresponds to a timed 4-meter walk, scored from 4 (walk completed less than 4.82 s) down to 0 (the participant was unable to complete the walk).
  The total score obtained on these three mobility tests ranged from 0 to 11, with higher values indicating greater mobility function.
-*Sensory abilities*, particularly visual acuity and hearing were assessed with a three-point Likert-type scale, ranging from 0 to 2 (where 0 corresponds to the highest sensory loss). Thus, sensory scale ranging from 0 to 4 with higher scores indicating better sensory functions.

Finally, the *self-reported exhaustion* dimension of frailty was assessed by evaluating perceived health condition (Short Form Questionnaire SF-36 with its two subscores for the physical and the mental health, (Ware and Sherbourne, 1992); and the General Health Questionnaire, GHQ-28, Sterling, 2011), self-reported cognitive complaint (Cognitive Difficulties Scale, CDS - McNair and Kahn, 1983) and routinization preferences (*french Routinization Scale–*RS^3^; Bouisson, 2002).

Overall, the participants presented a reduced capacity in several dimensions (physical, psychological, functional). This contributes to frailty and characterizes an increased vulnerability to stressors, without the presence of any concomitant neurological disease (Kelaiditi et al., 2013 for review).

The participants were then randomly divided into two groups matched according to the above-presented measures (see Table 1). One group was provided with HomeAssist and the other group was a control group (who were equipped of fake paper-based sensors).

**Table 1:** Participants' characteristics for control and equipped group.

**Old participants**	**Equipped group (*N* = 16) *Mean (SD)***	**Control group (*N* = 16) *Mean (SD)***	**Group comparison**
Age	80.38 (1.52)	82.88 (1.61)	*p* > 0.200
Gender	4 males	4 males	
Family Status	15 widowed/1 single	16 widowed	
MMSE _[0−30]_	27.81 (0.38)	27.56 (0.55)	*p* > 0.700
MNA _[0−30]_	24.13 (0.50)	23.88 (0.45)	*p* > 0.700
Body/Lean Mass Value	4.62 (0.18)	4.08 (0.33)	*p* > 0.150
Perceptive status _[0−4]_	2.69 (0.28)	2.75 (0.19)	*p* > 0.800
Physical status _[0−11]_	9.00 (0.43)	8.33 (0.86)	*p* > 0.400
Static Balance Testing	3.37 (0.24)	3.06 (0.40)	*p* > 0.500
[0-4]
Timed Get Up and Go Test	2.19 (0.19)	2.20 (0.23)	*p* > 0.900
[0-3]
Gait Speed Test [0-4]	3.44 (0.20)	3.07 (0.29)	*p* > 0.300
Perceived Health			
SF-36 physical [0-100]	58.78 (5.86)	52.84 (5.42)	*p* > 0.400
SF-36 mental [0-100]	68.12 (5.06)	66.30 (4.80)	*p* > 0.700
GHQ-28 [0-84]	19.87 (3.42)	20.69 (2.61)	*p* > 0.800
CDS _[0−148]_	30.97 (3.85)	43.93 (6.66)	*p* > 0.100
EPR _[0−40]_	15.68 (1.57)	15.81 (1.56)	*p* > 0.900

*MMSE, Mini Mental State Examination; MNA, Mini Nutritional Assessment; SF-36, Short Form-36; GHQ-28, General Health Questionnaire; CDS, Cognitive Difficulties Scale; EPR, Echelle de Préférence de Routinisation*.

All 32 caregivers were home care professionals, and all were female (with at least 1 year of home care experience and at least 6 months care giving for the older participant). Their tasks mainly involved providing support for domestic tasks, purchases and administrative tasks. Caregivers visited the older adults' homes anywhere between twice a month and once a day, depending on the care-receiver's difficulties.

According to the Helsinki convention, older participants gave their written informed consent before taking part in the study and local CPP^4^, CNIL^5^ and COERLE^6^ agreements were obtained.

### HomeAssist

**Figure 1:**
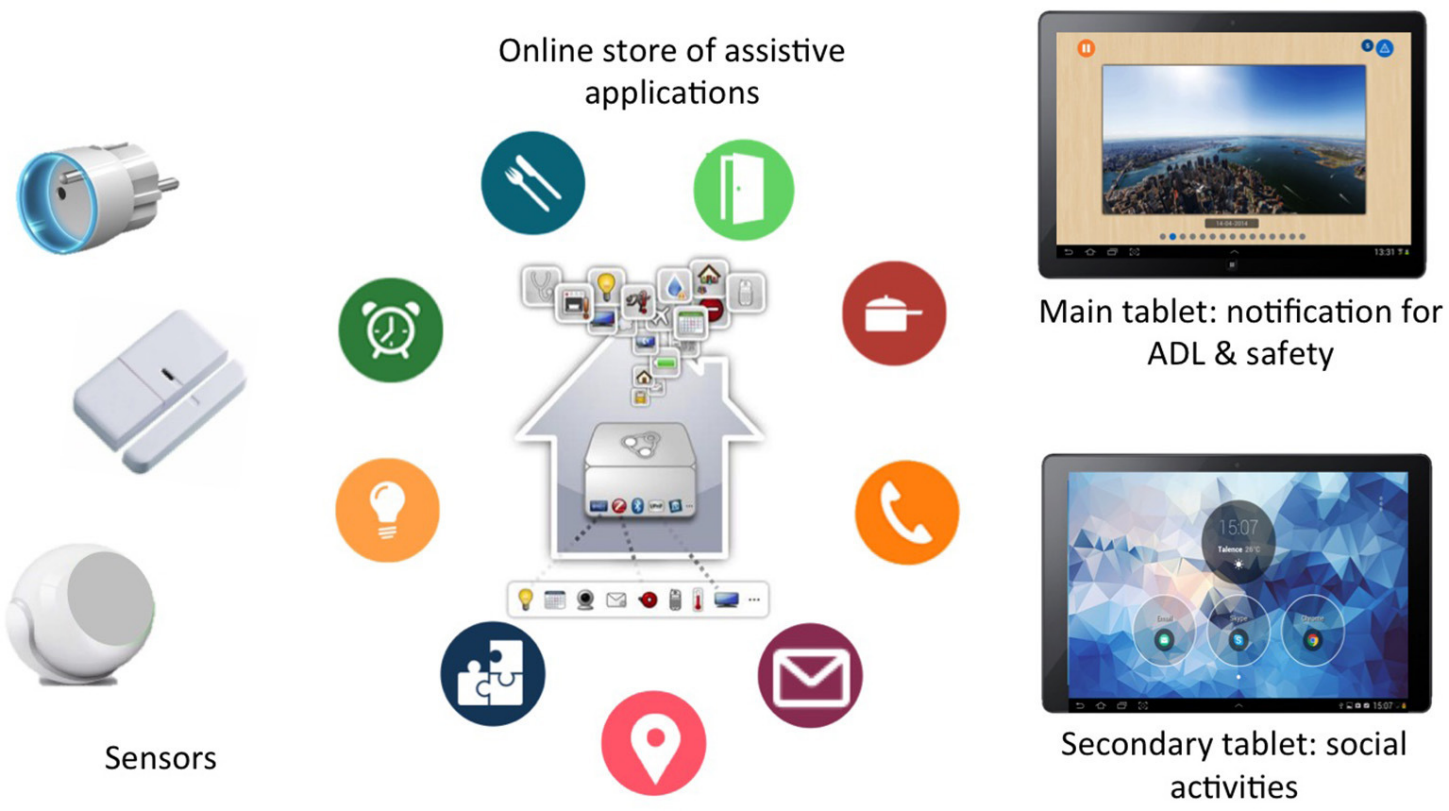
Examples of assistive applications from our online catalog.

We based the design of our assisted-living platform on previous human-centered AAL research (Dupuy et al., 2016, Consel et al., 2015), and implemented it with a set of wireless sensors and two touchscreen tablets. HomeAssist provides assistance in each of the three needs domains (Aguilova et al., 2014, Dupuy et al., 2015) thanks to an online catalog of assistive applications. This allows the assistive support to evolve with the user: new applications can be installed or deleted, depending on the user's needs. The assistive applications we supplied are described hereafter (see examples in Figure 1).

#### Applications for everyday activities

Thanks to sensors located in different parts of the user's home, the platform was able to monitor ADLs (in particular, getting up, meal preparation, toileting, dressing, and going to bed, Caroux et al., 2014). Omissions could be signaled by a reminder displayed on the main tablet. Also, this tablet could remind users of appointments or special events (birthdays, private and family events) thanks to an online calendar, also accessible to caregivers.

#### Applications for safety

To prevent falls during night, users were provided with a light path. A small sensor detected when users switched on their bedside light, which automatically activated the light path. Second, the front door was monitored by sensors, and an alert was triggered whenever the door was left open and unattended. Finally, electric appliances (such as stoves) were also monitored and could be automatically switched off. In critical situations, a text message was sent to the caregiver.

#### Applications for social participation

A dedicated tablet provided a simplified mailing system, which allowed messages to be sent using the voice alone (messages could be voice recorded) and a speech synthesizer to read messages out loud (Caroux et al., 2017). Also, users were provided with video telephoning and collaborative gaming apps (which the user could choose). Finally, users were informed of any social events organized by the town council.

#### Interaction support

**Figure 2:**
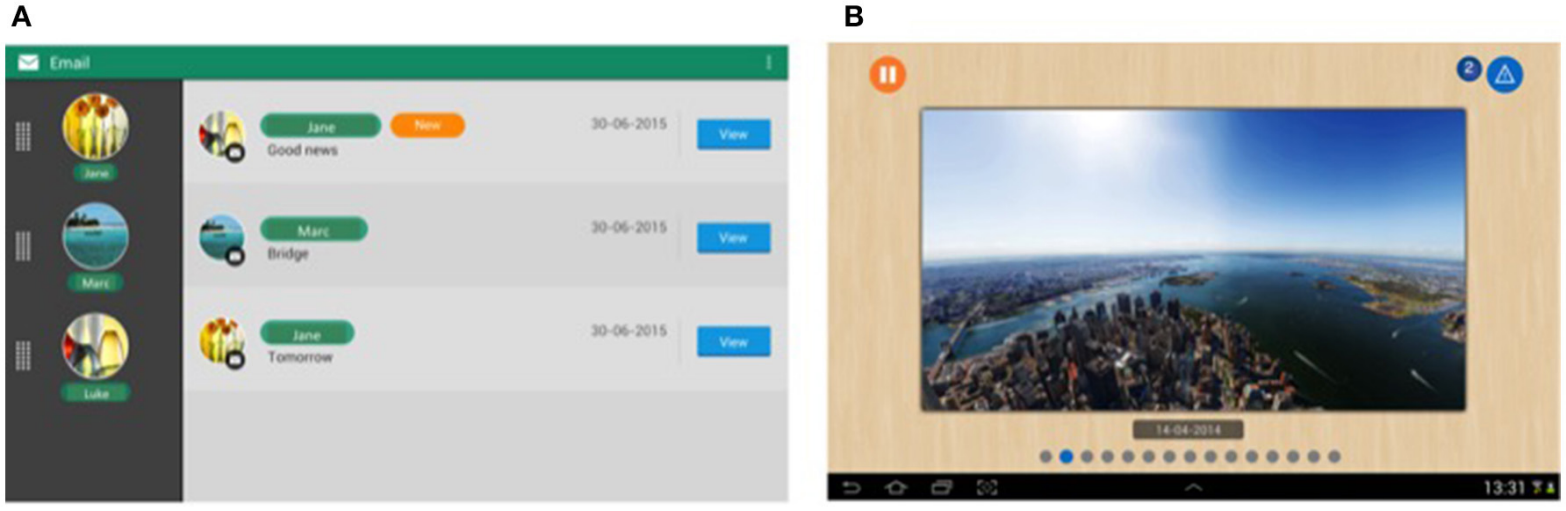
Snapshots of interfaces displayed on the tablets. **(A)** Inbox interface of our simplified mailing application; **(B)** Digital picture frame (from Dupuy et al., 2016).

Tablets have been shown to be easy to use for older adults (e.g., Fisk et al., 2009). Thus, following guidelines for older adult populations (e.g., ISO/TR 22411^7^. Fisk et al., 2009), we designed two tablets for our older users (Figure 2).

The main tablet was dedicated to sending notifications to the user about everyday activities and safety applications. This tablet was stationary, plugged into a power outlet, and centrally located in the user's home. It should be pointed out that notifications from the different applications were unified: heterogeneous assistive services notified users homogeneously. More precisely, we defined two categories of notifications: non-critical and critical, depending on the urgency of the message. This differentiation has been shown to be well understood and accepted by older users (Consel et al., 2015). Another feature of this tablet is that when it is left idle, it turned into a digital picture frame, for a shared experience with family and friends (Figure 2B). The second tablet was dedicated to social participation.

We chose to use two tablets to separate the two types of interactions for several reasons. First, since the main tablet functioned as a signaling device, it was switched on at all times and left in the same place, in order for it to be located and controlled rapidly (much like a landline telephone); the second tablet, however, did not provide any urgent information and thus could be mobile and switched off. A second motivation for using two tablets concerns cognitive cost. Indeed, if a user is using the tablet (using the email system for example) and an alert is displayed, they would have to switch to another task. However, task switching is an ability that has been shown to decline with age (e.g., Fisk et al., 2009). Thus, to reduce the cognitive cost related to task switching, we preferred to separate the interactions between two tablets. Finally, from a learning perspective, and considering age-related cognitive decline, having two interaction supports enables the trainer to gradually introduce the different functionalities (e.g., Fisk et al., 2009).

As for the deployment process, a home automation specialist installed the platform in the users' homes, and the sensors and tablets were positioned on the person's electrical appliances (e.g., coffee machine, toaster, bedside light) according to their routines, as analyzed by an occupational therapist. Once the installation process was complete, the older users and their caregivers followed 4 training sessions (once a week for a month) to help them understand and master the various functionalities of HomeAssist. More specifically, training sessions lasted approximately 1 h and comprised short exercises to learn to use the different assistive applications. Additionally, a concise paper-based manual was provided throughout the 6-month experiment, and users could call the research team 24/7 if they had a question or if the equipment malfunctioned.

### HomeAssist use

The HomeAssist uses and usages, as well as elicited satisfaction, were evaluated in the equipped group across the 6 months of platform usage. Three different measurements were carried out for assessing the HomeAssist usability, the related-user experience and user satisfaction, respectively: Time-based usage scenario tests, Attrakdif questionnaire (Hassenzahl, 2004) and the QUEST questionnaire (Demers et al., 2002).

Inspired by the timed-IADL assessment (Owsley et al., 2002), the Time-based usage scenario tests measured the user's performance in terms of effectiveness (accuracy of interaction behaviors) and of efficiency (interaction duration). Indeed, the score depends on the type and number of errors made by the user, and whether the task was performed within the allocated time (varying with respect to task difficulty). Precisely, these tests consisted of four everyday usage scenarios. Two tests were based on the main tablet and involved a critical notification (i.e., simulated door alert) and a noncritical one (i.e., simulated activity reminder). Two others scenarios addressed the use of the secondary tablet with one scenario related to a video telephoning and one other related to an e-mail tool (specially designed for older adults) (Caroux et al., 2017). These four usage scenarios were selected from the deployed services because all the participants choose them. The measures were collected at 6 weeks and at 6 months after the HomeAssist installation. Results from usage scenarios revealed that our participants performed very well (averaged score over 2.65 on a scale from 0 to 3), but they were more proficient with time for the two scenarios related to the main tablet, compared to the scenarios related to the secondary tablet (for detailed results, see Appendix 1 in Supplementary Material). Regarding user experience, participants had a positive experience (averaged score over 1.2 on scale from −3 to +3) improving with time (averaged score of 1.71). This improvement with time was greater for 4 dimensions (ergonomic quality, hedonic quality, appealingness, and anxiety) compared to the dimension of safety perception that started relatively high (averaged score of 1.01) and slightly increased across time (averaged score of 1.22) (for detailed results, see Appendix 1 in Supplementary Material). Finally, QUEST performances revealed that user satisfaction was very high (averaged score of 4.38 on a scale from 0 to 5), but slightly diminished between the 6th week (averaged score of 4.53) and the 6th month of HomeAssist use (averaged score of 4. 23).

Overall, these results on HomeAssist use indicated that the equipped participants were proficient in using assistive services, and they exhibited a positive user experience as well as user satisfaction.

### Measures

Assessments were performed twice for both the control and the equipped group: once before the beginning of the experiment (at t_0_), and again 6 months later (at t_6_). Indeed, the objective was to investigate the outcome of using HomeAssist, both in terms of benefits for the users' autonomy and in terms of caregiver burden. All caregivers underwent the same assessments. The following measures were collected.

#### Older participants' functional status

The *IADL Scale* (Lawton et al., 1982) for a self-assessment: it consists of a 24-item scale on ADL abilities, with a scoring based on a 5-point Likert-type format, ranging from 0 (not at all difficult) to 4 (very difficult), so that the total score ranged from 0 to 96. To give an example, one of the items of this scale is: “For you, eating is: Very difficult (4)–Not at all difficult (0).”

The *IHVA Scale* for a caregiver assessment: it was completed by each participant's caregiver to collect their perception of the care-receiver's functional status. Four-point Likert-type items, ranging from 0 (the care-receiver never does it) to 3 (always does it) compose this 12-dimension questionnaire. Each dimension comprises 10 items, giving scores that range from 0 to 30, with higher scores indicating greater everyday abilities. As an example, an item of this questionnaire is: “Buys medicine and takes it as recommended by the prescription: never does it (0)–always does it (3).”

#### Caregiver burden

*The MBI scale*: all caregivers underwent the MBI, which assesses three aspects of professional burden: emotional exhaustion (i.e., the feeling of being exhausted by one's work), depersonalization (i.e., an unfeeling and impersonal response toward recipients of one's care), and personal accomplishment (i.e., the feeling of competence and success), on a 7-point Likert scale ranging from 0 to 6 (higher scores for the two first dimensions, and lower scores for the third, indicate a higher degree of burnout). To illustrate this, one of the items of this scale is: “Do you feel worn out at the end of the working day: Never – Always.” As proposed by Ahola et al. (2014), we computed a global burnout score using the following formula: [0.4^*^exhaustion + 0.3^*^depersonalization + 0.3^*^(48 – personal accomplishment)]. Thus, higher scores indicate a higher degree of experienced burnout. This inventory assessed global professional burnout, not only the burnout related to caring for the participant of the study, that is why we also administrated an other scale.

The *IADL support scale*: it is an adaptation of the Lawton scale presented earlier, to assess burden for IADL support. Thus, answers varied from 0 (very easy to assist), to 4 (very hard to assist), in reference to the assistance given to the participant in particular. For instance, an item of this scale is: “For you, the support that you provide for eating is: Very hard–Very easy.”

### Statistical analyses

For assessing the outcomes of using HomeAssist, we compared our equipped and control participants before and after the intervention. Thus, mixed ANOVAs have been performed with the following statistical design: Time as an intra-individual independent factor with two modalities (t_0_ vs. t_6_), and Group as an inter-individual independent factor with two modalities (equipped vs. control) on the functional status and caregiver burden measures presented above. When a Time^*^Group interaction effect was obtained, *Student t-tests* were performed for each group. Additionally, *Levene tests* were previously performed to ensure the homogeneity variance of all data collected. For plotting data, z-scores are computed for each measure. The raw scores for each measure are presented on Appendix 2 in Supplementary Material. The statistical analyses were done using SAS SPSS Statistics 22.

## Results

### HomeAssist effect on older adults' functional status

**Figure 3:**
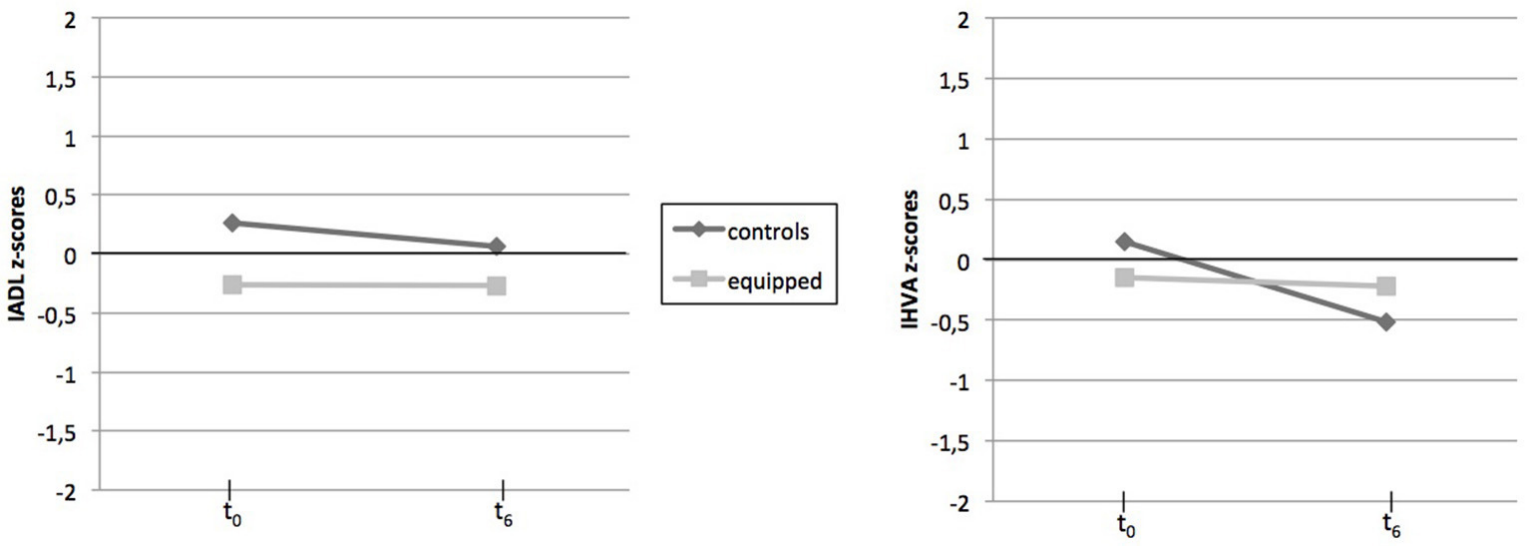
Pre- and Post-intervention z-sores of functional status measures for each group. M, mean; SD, standard deviation, IADL, Instrumental Activities of Daily Living; IHVA, Inventaire des Habilités pour la Vie en Appartement.

Z-scores of the two measures of functional status are plotted on Figure 3.

Concerning self-perception of everyday difficulties by older adults themselves (IADL scale), no significant effect was found, either for the Time and Group factors or for the Time^*^Group interaction effect (*p* > 0.100).

Regarding the perception by caregiver (IHVA scale), an effect of Time was observed [*F*_(1, 30)_ = 24.53; *p* < 0.001; η^2^ = 0.45], with overall everyday abilities reported by caregivers as being lower after the 6-month experiment. No effect of Group was observed (*p* > 0.100). However, a strong Time^*^Group interaction effect was revealed [*F*_(1, 30)_ = 15.70; *p* < 0.001; η^2^ = 0.34]. Results indicate that the functional status of control participants decreased considerably over time [*t*_(1, 15)_ = 4.69; *p* < 0.001; η^2^ = 0.59], whereas no significant decline was observed for equipped participants (*p* > 0.100).

We can point out that the adding of the living place factor (i.e., population density according the urban vs. rural distinction) has not changed the above result on the IADL or the IHVA scores (with *p*-value > 0.100 for Living place effect or its combined effects with Time and Group factors).

Overall, perception of functional status by older adults did not differ over time, for both control and equipped participants. However, according to caregivers, equipped participants were perceived as more autonomous compared to controls.

### HomeAssist effect on caregiver burden

**Figure 4:**
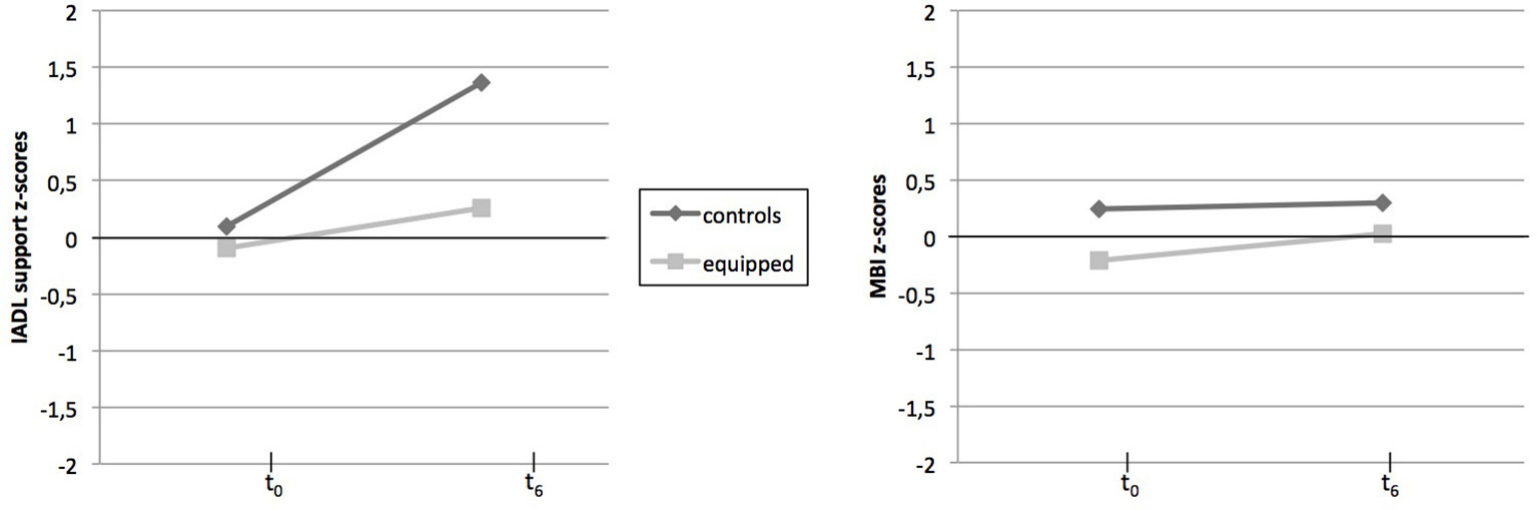
Pre- and Post-intervention z-sores of caregiver burden measures for each group. M, mean; SD, standard deviation, IADL, Instrumental Activities of Daily Living; MBI, Maslasch Burnout Inventory.

Z-scores of the two measures of caregiver burden are plotted on Figure 4.

Concerning MBI scores, a global effect of Time [*F*_(1, 30)_ 4.69, *p* = 0.04; η^2^ = 0.13] was revealed, with a higher perceived burnout after 6 months for both groups of caregivers. No significant effect of Group or of Time^*^Group was observed (*p* > 0.100).

Regarding the caregiver's version of the IADL scale, results showed significant effects: a strong effect of Time was observed [*F*_(1, 30)_ = 22.98; *p* < 0.001; η^2^ = 0.43], with an increase in caregiver burden over time. No effect of Group was observed (*p* > 0.100). Moreover, the Time^*^Group interaction effect was significant [*F*_(1, 30)_ = 7.12; *p* = 0.012; η^2^ = 0.19], with a large increase in caregiver burden for the control group between t_0_ and *t*_6_[*t*_(1, 15)_ = −4.74; *p* < 0.001], but no significant increase [*t*_(1, 15)_ = −1.72; *p* = 0.10] for the equipped group.

We can point out that the adding of the living place factor (i.e., population density according the urban vs. rural distinction) has not changed the above result on the MBI, as well as the caregiver's IADL score.

To sum up, a time-related increase was observed for both overall professional burnout (MBI) and caregiver burden related to supporting the older participant (IADL support scale). Yet, the evolution of caregiver burden related to sustaining our participants in their ADL was slower in the equipped group compared to the control group.

## Discussion

This study aimed to assess the benefits provided by HomeAssist on FIs' functional status and on caregiver burden.

The first major result concerned measures of functional status. Results showed that no significant difference was reported by older adults between t_0_ and t_6_, irrespective of group conditions (equipped vs. control). On the contrary, according to caregivers, they perceived no changes in everyday functioning in the equipped group, whereas everyday functioning deteriorated in control participants. In other words, the HomeAssist intervention protected the FIs from functional losses. Thus, the present results are in line with research by Tomita et al. (2007) and by Vincent et al. (2006), who showed that their AAL technologies also helped to slow functional degradation. Therefore, it appears that AAL systems are promising interventions for reducing the pace at which functional losses occur, especially in the case of FIs as reported in the present study. This assumption is consistent with previous studies showing that environmental support can be a fruitful approach for helping FIs to perform ADLs, or even for reducing the progression of functional degradation (e.g., Mann et al., 1999). By extension, this supports the recent theoretical frameworks promoting an adaptation of the Environmental Support Hypothesis (Craik, 1986) for the conception of assistive functionalities for older adults (Morrow and Rogers, 2008), as well as for those with dementia (e.g., Gillespie et al., 2012).

The discrepancy between older adults' self-perceived scores and those attributed by their caregivers deserves consideration. Several explanations can be advanced. First, several studies have shown that aging is associated with a decline in the ability to accurately estimate everyday difficulties (Gold, 2012). This highlights the importance of questioning an informal or professional caregiver when assessing older people's difficulties. Second, two distinct scales were used to assess everyday functioning according to the participant's role in the study (older adult vs. caregiver). Thus, potential differences in the psychometric properties of each scale probably contribute to the care-receiver—caregiver discrepancy. In other words, using the IHVA with its 120 items for care-receivers could be more appropriate for stressing an impact in everyday functioning.

Overall, based on caregivers' reports, this study is the first whose results agree with the expected outcome of preventing older adults' functional losses, in the case of frail individuals and a multitask-based AAL intervention.

Regarding caregiver-related measures, three results can be highlighted. First, significant negative effects were observed over time, from t_0_ to t_6_, on the MBI and the IADL support scales. Such observations are consistent with previous findings showing that caregiver burden increases with care experience (Zarit et al., 1986; Ahola et al., 2014). Classically, this increased burden with time is explained by tedious working conditions, particularly when care is performed at home (From et al., 2013). Furthermore, the time-related loss in everyday functioning in older participants may also explain the increase in caregiver burden. Indeed, according to the caregivers themselves, everyday functioning decreased over time for their care-receivers, thus increasing the caregivers' workload. The relationship between caregiver burden and the functional health of the care-recipient is well known in the geriatric literature, notably thanks to studies focusing on the Stress Process model (Pearlin et al., 1990). Such a relationship has been clearly demonstrated for FIs (Lu et al., 2016).

Second, regarding MBI scores as an indicator of subjective professional burden, no effect (including the group effect) reached significance. As a reminder, the MBI assesses perceived psychosocial health at work in terms of emotional exhaustion, depersonalization, and personal accomplishment. Thus, HomeAssist did not significantly reduce caregivers' subjective feelings of professional burden.

Thirdly, and importantly, the assessment of the discomfort of caregivers for supporting IADL showed significant differences over time between equipped and control participants. Results indicated that the increase of burden across time is slighter in the equipped group, highlighting the positive impact of HomeAssist on caregivers' workload for supporting the everyday functioning of their care-recipients.

Taken together, the two latest results suggest that HomeAssist efficacy was more tangible for objective dimension of professional burden compared to its subjective dimension. This conclusion mirrors some findings relative to the distinction between objective and subjective burden of caregivers (Montgomery et al., 2000). Indeed, objective burden refers to perceived infringement on or disruption of tangible aspects of the caregiver's life. As stressed by Auer et al. (2015), the objective burden is related to the symptom gravity of care recipient, such as functional losses or behavioral problems. By contrast, subjective burden refers to both the extent to which the caregiver perceives care to be overly demanding and the emotional impact of caregiving missions. The subjective burden is often assigned to both internal individual factors (i.e., personality, life experience, motivation, attitude toward care receivers, education) and organizational factors (i.e., work conditions such as lifting heavy individuals, time stress, etc.).

In light of the two previous studies, which showed a marked reduction of caregiver burden (Magnusson and Hanson, 2005; Vincent et al., 2006), the slowed progression of caregiver burden observed in the present study could be explained by several factors. First, the present study involved professional caregivers, instead of family or informal ones. The caregiver's presence at recipient home is less long for professional caregiver, compared to family ones who often are the spouse of frail individual. Consequently, the opportunities to experience the AAL tools benefits for care activities are probably lower for professional careers than family one. In other words, the strong home presence of family carers likely ensures an increased accuracy in the estimation of AAL efficacy for recipient, and in turn for caregiver. In a connected way, a second explanation relies on the measurement method. As we included professional caregivers, the MBI has been used for assessing professional burden while the Zarit scale or “hand-made” interviews have been administered in other studies for a family burden purpose. A final factor could be the very nature of the AAL tool: our platform was primarily designed to meet frail older adults' needs rather than caregivers' ones. Consequently, the benefits for caregivers are primarily indirect and related to objective dimension of their burden. An AAL tool that focuses more on caregivers' needs could achieve the expected outcome of reducing their burden.

### Limitations

Despite encouraging results, some limitations deserve to be mentioned. First, we can notice the small size of studied sample, due to the real-life setting of a field study and the associated financial constraints for affording HomeAsssit to each participant. A larger sample would statistically provide a more powerful generalization of the present results. However, the present pilot study is a required step before a more robust study with an extended sample.

Second, all the studied measures have not been collected according to double blind method. Indeed, it seems difficult to envision an experimental design where raters would not able to distinguish equipped participants from controls. Consequently, a potential risk of inflated effect of HomeAssist on measures cannot be totally excluded. Hence, our results are more promising results (Level 2 on the four-level scale) than effective (Level 3) according to the evidence-based public health (EBPH) typology for classifying interventions' study by level of scientific evidence (Brownson et al., 2009).

Thirdly, as done by a large range of aging studies, subjective ratings are collected to probe burden and everyday functioning with the potential well-known risk of responses biases. One could argue that the caregivers' perceptions may be positive regarding to HomeAssist, and thus its benefits may be over-estimated. In a previous work, we have compared Assistive Technology (AT) needs expressed from old adults to their professional caregivers (Dupuy et al., 2015). Clearly, caregivers were more accurate than care recipients i.e., their perceived AT needs were significantly correlated with cognitive or physical losses exhibited by the care recipients while such correlations failed to be found for AT needs expressed by the care recipients. Thus, a response bias is not totally excluded, albeit not likely.

Four, the 6-month duration of HomeAssist intervention could be limiting for accurately probing the long-term AAL effects on everyday functioning. Indeed, a longitudinal follow-up over 12 months is frequently reported for demonstrating responsiveness to a non-drug intervention on everyday functioning amongst older samples (Reijnders et al., 2013). Consequently, a longer intervention period may increase its health outcomes in terms of FIs' everyday functioning, as well as of burden of professional caregivers.

Five, it would have been interesting to highlight the benefits of using HomeAssist, by including an experimental condition entailing only a single-task assistive technology. Such condition would provide a comparison between our multiple-task intervention and a single-task intervention, as promoted in a silo-based approach. Future studies built with this kind of experimental design would be able to provide a selective and analytical assessment of AAL-based environmental interventions.

Despite the above-mentioned limitations, the present study has the advantage of deploying in a real-life setting a multi-task AAL platform for six months, and assessing the outcomes both on FIs' autonomy and caregiver burden.

## Conclusion

This pilot study presented a multi-task platform, designed to bring assistance in everyday activities, safety, and social participation. The main results showed positive outcomes in terms of evolution of functional status in FIs and the objective dimension of caregiver burden. Therefore, for the first time, an AAL is showed to provide functional benefits for both FIs and professional caregivers.

Importantly, this study is the byproduct of the HomeAssist project, which has been using a human-centered approach for developing all the facets of the platform. In particular, an ability-based design was used (Wobbrock et al., 2011) for developing assistive services. The features of the platform's user interactions were empirically validated to assess the efficiency of the services in delivering their assistance. Additionally, self-determination-based design was introduced as motivational leverage for HomeAssist acceptance. As a result, the present study yielded to a high HomeAssist-related acceptance, as well as a highly positive experience and satisfaction amongst our frail participants. Moreover, after the 6 months of follow-up, only two participants wished to quit using our AAL, while the remaining 14 kept it.

From a wider perspective, the present study demonstrates the feasibility of designing and deploying a multi-task platform for FIs in real setting, while conducting a controlled experimental study. AAL devices offer a new research avenue for moving forward the field of environmental gerontology, by providing new opportunities for an appropriateness of services for coping with the functional losses of FIs, and thus promoting aging in place.

## Author contributions

LD took part of the design of the work, acquire, analyse and interpret data, and write the research article. CF took part of the analysis and interpretation of data. CC took part of the supervision of computer research related to HomeAssist platform and give a final approval of the version to be published. HS took part of the design and research supervision of the work, of the analysis and interpretation of data, the redaction and revision of the research article, and give a final approval of the version to be published.

### Conflict of interest statement

The authors declare that the research was conducted in the absence of any commercial or financial relationships that could be construed as a potential conflict of interest.

## Footnotes

^1^In community-dwelling elderly populations, frail and pre-frail individuals represent around 40% of people aged over 65.

^2^First, the Body Mass Index (BMI) was calculated according to the standard formula [BMI = mass (kg)/(height (m))^2^]. The BMI is scored from 0 to 3 with higher values indicating higher BMI values. Second, the brachial and calf perimeters are scored from 0 to 2 with a higher value indicating higher lean mass value. Summed, the two indices provide a score from 0 to 5, with a higher score indicating a better body mass.

^3^RS is a French analog of the Routinization scale of Reich and Zautra (1991).

^4^CPP: Comité de Protection des Personnes Sud-Ouest et Outre mer (in english, Committee for Person Protection for Aquitania and french oversea ‘s territories).

^5^CNIL: Commission Nationale de l'informatique et des libertés (the French national committee for information and communication technologies and privacy Rights of persons).

^6^Ethics committee of the National French Institute of Informatics and Mathematics (Inria).

^7^International Standard ISO/TR 22411: Ergonomics data and guidelines for the application of ISO/IEC Guide 71 to products and services to address the needs of older persons and persons with disabilities (2008).
