# Supplementary material for: Everyday Functioning Benefits from an Assisted Living Platform amongst Frail Older Adults and Their Caregivers
Source: Front Aging Neurosci. 2017 Sep 28;9:302. doi: 10.3389/fnagi.2017.00302 (PMC5626945; doi:10.3389/fnagi.2017.00302)
Supplement: Supplementary file 1 [file DataSheet1.pdf]

Everyday Functioning Benefits From an Assisted Living Platform Amongst Frail Older Adults and Their Caregivers

Authors: Dupuy, L.[1,2,*], Froger, C.[1], Consel, C.[3,1] & Sauzéon, H.[1,2,*]

Appendix 1
Measures related to the HomeAssist use

- *Usage scenario tests* : Each usage scenario test ranges on a scale of 3; the score depends on the type and number of errors made by the user, and whether the task was performed within the allocated time (varying in respect of task difficulty). A score of 3 denotes a task performed without errors and within the allocated time. A score of 2 corresponds to a task performed with minor errors but within the allocated time. A score of 1 is when the user did not complete the task or made major errors. Participants underwent these tests twice, i.e. 6 weeks and 6 months after the HomeAssist installation (modalities of Time factor). The Table 2 denotes the means, the standards deviations, and the main statistical results from the two ANOVAs performed for assessing time effect and usage scenario factor. Overall, the improvement of interaction behaviors across time was observed for all the two scenarios related to the main tablet while a slight converse effect was observed for all the two scenarios related to the secondary tablet (significant interaction effect between Time and scenario factors).

	**Usability Score [0-3]**	
	t6w	t6m
Door alert- Main tablet	**2.52 (0.81)**	**3.00 (0.00)**
Activity reminder –Main Tablet	**2.75 (0.68)**	**2.87 (0.51)**
Video telephoning -2nd Tablet	3 .00 (0.00)	2.81 (0.50)
E-mailer -2nd tablet	3.00 (0.00)	2.87(0.54)
**ANOVAs results**		
Time effect	F(1,15)=0.33; ns	
Scenario effect	F(3,45)= 0.74; ns	
Time*Scenario	F(3,45)= 3.12; p<0.4 ; η^2=.172	

Table 2. Mean (M) and standard deviation (SD) sores from the four usage scenarios according to time condition (t6w: 6 weeks after; t6m : 6 months after HomeAssist installation).

- *The Attrakdiff questionnaire* decomposes user experience into five dimensions: ergonomic quality, hedonic quality, appealingness, anxiety and safety perception, and social influence. Answers to the questionnaire range over a scale of 7 points, from −3 to 3, including two antonyms (e.g., nervous/relaxed). Each dimension of the questionnaire consists of 6 items. Participants underwent this questionnaire tests three times, i.e. before the HomeAssist installation (baseline for participant's Perceptions regarding Assistive technologies), 6 weeks and 6 months after the HomeAssist installation (experimental conditions for participant's perception about HmeAssist). These three times refer to the modalities of Time factor. The Table 3 denotes the means the standards deviations, and the statistical results from the ANOVA performed for assessing the time effect and the user-experience dimensions effect, and their combined effect. Overall, the improvement of user experience across time was observed for all the dimensions, except for safety perception remaining nearly equal across time (significant interaction effect between Time and Dimension factors).

	**User experience scores [-3; +3]**		
	t0	t6w	t6m
Ergonomic quality	**0.57 (0.20)**	**0.92 (0.33)**	**1.52 (0.22)**
Hedonic quality	**0.71 (0.23)**	**0.95 (0.20)**	**1.68 (0.22)**
Appeallingness	**0.89 (0.31)**	**1.58 (0.30)**	**2.00 (0.28)**
Anxiety	**0.41 (0.25)**	**1.60 (0.28)**	**2.13 (0.17)**
Safety	1.01 (0.13)	1.12 (0.17)	1.22 (0.21)
**ANOVA Results**			
Time effect	$F(2,30)= 7.03; p <.01 ; \eta^2=.319$		
Dimension effect	$F(4,60)= 4.50; p <.01 ; \eta^2=.234$		
Time*Dimension effect	$F(8,120)= 3.50$ $p =.001 ; \eta^2=.189$		

Table 3. Mean (and standard deviation) sores from the five dimensions of user experience measured with the Attrakdif questionnaire according to time condition (t0: before HomeAssist installation; t6w: 6 weeks after; t6m : 6 months after HomeAssist installation).

- *The QUEST 2.0 questionnaire* is a measure of user satisfaction with assistive technologies with two components, Device and Services. The questionnaire consists of 12 items and each item is evaluated on a scale ranging from 1 to 5. A value 1 corresponds to 'not satisfied at all' and a value 5 to 'very satisfied'. The total score is on a scale of 5. A high score denotes a high user satisfaction with the technology. Participants underwent this questionnaire twice, i.e. 6 weeks and 6 months after the HomeAssist installation (modalities of Time factor). The Table 4 denotes the means, the standards deviations, and the main statistical results from the ANOVA performed for assessing the time effect and the user-experience dimensions effect. Overall, the user satisfaction with HomeAssist was high, but slightly decreased with time (significant time effect), and assistive services elicited more satisfaction than devices used for HomeAssist (tablets and supports) (significant dimension effect).

	**User Satisfaction scores [0-5]**	
	t0	t6w
**Device**	4.24 (0.45)	3.97 (0.48)
**Assistive Service**	4.82 (0.31)	4.50 (0.59)
**ANOVA Results**		
Time effect	$F(1,15)= 7.43$; $p <.02$; $\eta^2=.331$	
Dimension effect	$F(1,15)= 17.48$; $p =.001$; $\eta^2=.538$	
Time*Dimension effect	$F(1,15)= 0.10$; ns	

Table 4. Mean (and standard deviation) sores from the two dimensions of user satisfaction measured with the QUEST 2.0 questionnaire according to time condition (t6w: 6 weeks after; t6m : 6 months after HomeAssist installation).

Appendix 2
Raw scores relative to pre- and post-measures of functional status of older participants and caregiver burden.

	**Equipped** *M (SD)*		**Control** *M (SD)*	
	t0	t6	t0	t6
**IADL** [0-96]	11.44 (2.19)	11.37 (2.36)	17.56 (3.41)	15.19 (3.29)
**IHVA** [0-360]	309.63 (6.78)	307.31 (6.49)	319.01 (8.93)	298.06 (9.55)
**MBI** [0-31]	19.05 (1.96)	20.12 (1.60)	21.00 (2.68)	21.41 (2.62)
**IADL support** [0-96]	6.62 (1.59)	9.06 (2.82)	7.94 (1.82)	16.50 (2.82)

Table 5. Pre and Post intervention raw sores of functional status and caregiver burden measures for each group. M: mean; SD: standard deviation, IADL: Instrumental Activities of Daily Living; IHVA: *Inventaire des Habiletés pour la Vie en Appartement*; MBI: Maslach Burnout Inventory.
